# A Study on the Corrosion Behavior of Fe/Ni-Based Structural Materials in Unpurified Molten Chloride Salt

**DOI:** 10.3390/ma18071653

**Published:** 2025-04-03

**Authors:** Unho Lee, Min Wook Kim, Jisu Na, Mingyu Lee, Sung Joong Kim, Dong-Joo Kim, Young Soo Yoon

**Affiliations:** 1 Department of Materials Science and Engineering, Gachon University, 1342, Seongnam-daero, Sujeong-gu, Seongnam-si 13120, Republic of Korea; uno94@gachon.ac.kr (U.L.); kimmw47@gachon.ac.kr (M.W.K.); skwltn991004@gachon.ac.kr (J.N.); acdc5640@gachon.ac.kr (M.L.); 2 Department of Nuclear Engineering, Hanyang University, 222 Wangsimni-ro, Seongdong-gu, Seoul 04763, Republic of Korea; sungjkim@hanyang.ac.kr; 3 Materials Research and Education Center, Auburn University, 275 Wilmore Labs, Auburn, AL 36849, USA

**Keywords:** molten salt reactor, corrosion, structural material, stainless steel, nickel-based superalloys, Gibbs free energy

## Abstract

The molten salt reactor is a fourth-generation nuclear power plant considered a long-term eco-friendly energy source with high efficiency and the potential for green hydrogen production. The selection of alloys for such reactors, which can operate for more than 30 years, is a primary concern because of corrosion by high-temperature molten salt. In this study, three Fe- and Ni-based alloys were selected as structural material candidates. Corrosion immersion tests were conducted in NaCl–KCl molten salt for 48 h at 800 °C and 40% RH conditions in an air environment. In the absence of moisture and oxygen removal, ClNaK salt-induced damage was observed in the investigated alloys. The corrosion behavior of the alloys was characterized using various techniques, including scanning electron microscopy, energy-dispersive X-ray spectroscopy, X-ray photoelectron spectroscopy, and Auger electron spectroscopy. The results show that the corrosion process can be explained by salt-induced surface damage, internal ion migration, and depletion to the surface. The corrosion rate is high in SS316L (16Cr-Fe), N10003 (7Cr-Ni), and C-276 (16Cr-Ni), in decreasing order. Based on the corrosion penetration, ion elution, and interfacial diffusion results, C-276 and N10003 are good candidates for structural materials for MSRs. Therefore, Ni-based alloys with high Cr content minimize surface damage and ion depletion in unpurified molten salt environments. This indicates that Ni-based alloys with high Cr content exhibit highly corrosion resistance.

## 1. Introduction

**Figure 1:**
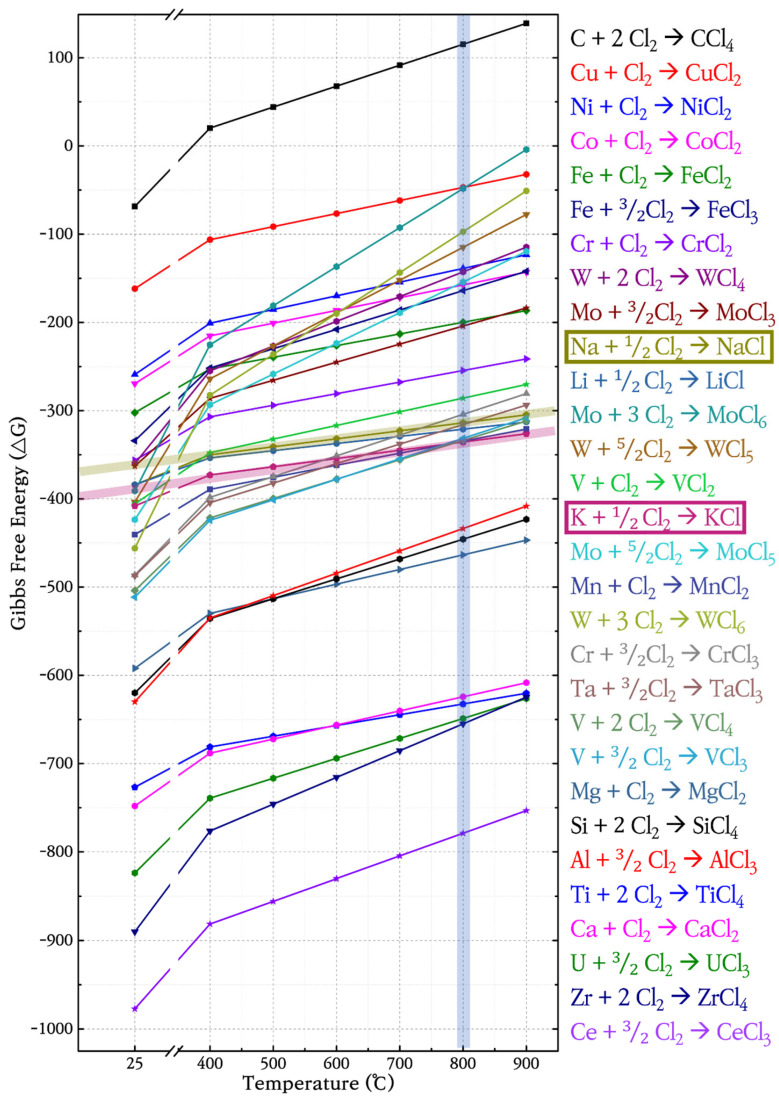
Gibbs free energies of chlorine compounds with ClNaK.

The social and cultural acceptance of nuclear energy has been hindered by safety concerns [1,2]. Considerable research has therefore been conducted on improving the safety of nuclear reactors. The safety measures investigated include human error exclusion [3,4], extreme-situation simulation [5,6], and functional enhancements such as the application of accident-tolerant fuel cladding [7,8], high-entropy alloys [9], and surface treatment for nuclear materials [10,11,12] to maintain reactor operation safety [13]. In particular, the Oak Ridge National Laboratory (ORNL) has proposed an unconventional concept for a novel, safe reactor called the molten salt reactor (MSR) [14]. MSRs are fourth-generation reactors that can use molten salt as a nuclear fission fuel. The fuel can be instantly solidified to stop the nuclear reaction in the event of an accident [15]. Previous MSRs are small modular reactors (SMRs) that use fluorine-based molten salt (FLiNaK) [16,17,18]. Because the low operating temperatures and solubility of uranium salts in such MSRs are insufficient to provide high power densities, a 400 MWt reactor employing chlorine-based molten salt was designed to overcome these disadvantages. Suitable structural materials are sought for this reactor. The use of Cl-based instead of F-based salt has the advantages of (1) high-energy operation due to the high solubility of the uranium salt, (2) It has a high operating temperature of 650 °C or higher, allowing the production of green hydrogen, and (3) simple reactor design with passive circulation by fast neutrons. The ability of the substrate to withstand the high-temperature behavior of metal–Cl compounds and its surface corrosion reaction in molten salt are critical considerations in the selection of the substrate material. Previous studies have indicated that Fe- and Ni-based alloys are promising corrosion-resistant alloys that are suitable as structural materials in MSRs [19,20]. In particular, studies are being conducted on the long-term use of stainless steel and Ni-based superalloys in molten salt environments for 30 years, owing to the high corrosion resistance of the passive surface Cr_2_O_3_ and Ni–Mo–Cr in these alloys. Their anti-corrosion properties were investigated using thermodynamic analysis in previous studies, which found that these materials rarely reacted with molten salt because of their stable surface energy [21,22]. The thermodynamics of the reactions between the alloys and molten salts during (1) surface corrosion, (2) ion elution, and (3) intergranular precipitation were analyzed. Thus, 800 °C molten ClNaK salt provides a ΔT of approximately 150 °C at the NaCl–KCl eutectic point of 657 °C. Figure 1 is a graph showing the Gibbs free energy in which each element forms Chloride according to temperature. The NaCl–KCl Gibbs free energy data in Figure 1 was used to select candidate structural materials comprising mainly Fe, Ni, Cr, and Mo. Three candidate alloys composed of these elements were immersed in ClNaK molten salt at 800 °C for 48 h and subsequently characterized to elucidate their mechanisms.

## 2. Materials and Methods

### 2.1. Structural Alloy

Stainless steel 316L (SS316L), Hastelloy C-276 (C-276), and Hastelloy N10003 (N10003) with the compositions shown in Table 1 (supplier provided) were purchased from Daeheung Welltech^®^ (Ulsan, Republic of Korea), an affiliate of Haynes^®^ (Westlake Village, CA, USA). All corrosion tests were conducted after cutting the metal into pieces that were 1 cm in width and 1 cm in length.

### 2.2. Preparation of Eutectic Salts and Structural Alloy Corrosion Test

NaCl and KCl-based eutectic salts were prepared at a specific molar ratio of 0.506 moles of NaCl and 0.494 moles of KCl to investigate the corrosion behavior of the structural materials. This specific molar ratio has a minimum eutectic point at 657 °C. Both NaCl and KCl (99.9% pure) were purchased as reagents from Duksan Chemical^®^ (Ansan, Republic of Korea). The mixed Cl-based salts were filled into a high-purity alumina boat (Purity, 99.8%, recrystallized aluminum oxide, nonporous). The SS316L, C-276, and N10003 structural material candidates were placed in separate crucibles to prevent galvanic corrosion in the substrate and reacted at 800 °C for 48 h in a muffle furnace (JB-30, KANTHAL, A-1 Molding, K-Type J-ONE Co., Ltd., Gunpo, Republic of Korea). The experiment was conducted at an average temperature of 20 °C (20 ± 2 °C) and relative humidity (RH) of 40 ± 10% under a standard air environment. The residual surface salt from each reacted structural material was dissolved in high-purity deionized water. The types and concentrations of the ions in the dissolved residual salt solutions were analyzed using inductively coupled plasma–mass spectrometry (ICP–MS; NexION 350D, Perkin-Elmer SCIEX, Framingham, MA, USA).

### 2.3. Surface and Cross-Section Treatment for Characterization

Epoxy treatment was performed on the alloys to obtain clean surfaces and cross-sections for optical analysis both before and after the corrosion reaction. SiC paper (180, 320, 600, and 900 grit) was used to grind each sample to adjust its thickness and roughness. The sample was then polished using a diamond suspension solution and 9, 3, and 1 micro pads for 7 min and subsequently washed using a colloidal silica solution (0.04 micron) for 30 min. The changes in its surface, cross-section, and grain boundaries were observed in micrographs obtained using optical microscopy (OM; HIROX, RH-2000, Oradell, NJ, USA) and scanning electron microscopy (SEM; Hitachi S-4700, Hitachi, Tokyo, Japan). Energy dispersive X-ray spectroscopy (EDX; Oxford, UK, 100 mm^2^, 127 eV) was performed to investigate the elements in the scanned surfaces and cross-sections. The element ratios and oxide layer thicknesses were determined using the element line scanning function of the SEM–EDX system. Atomic force microscopy (AFM; Park NX10, Resolution: 0.05 nm, Park Systems, Suwon, Republic of Korea) was performed to measure the change rates and thicknesses of the thin oxide film, protective layer, and substrate before and after corrosion. The surface corrosion layer was removed using Ar sputtering etching for X-ray photoelectron spectroscopy (XPS; K-alpha, Thermo Scientific, Waltham, MA, USA) depth analysis. Etching was performed from the alloy sample surface to its inner bulk so that quantitative and other elemental measurements could be performed to complement the SEM and AFM analysis results. Auger electron spectrometry (AES; PHI-710, KIST, ULVAC-PHI, Inc., Chigasaki, Japan) was performed to confirm the element ratio and binding energy. The surface, diffusion layer, and alloy inner bulk zone characteristics were cross-validated using the Spot XPS and EDX functions of the AES system.

## 3. Results

### 3.1. Analysis of Surface Morphologies and Corrosion Behavior

**Figure 2:**
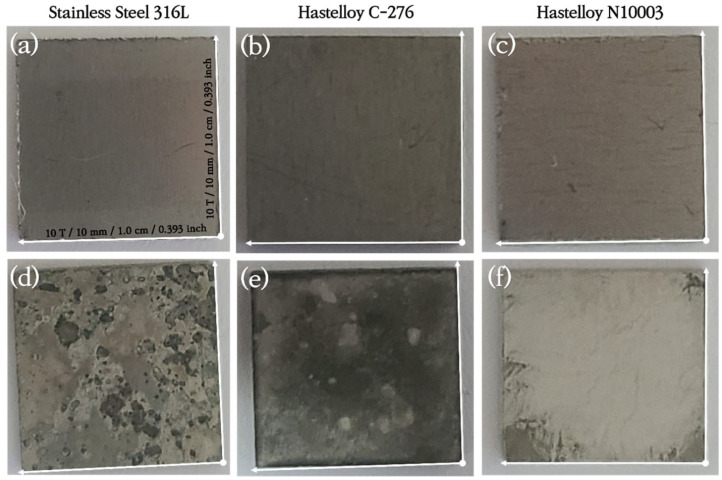
Images of structural materials (**a**–**c**) before and (**d**–**f**) after immersion in ClNaK salt for 48 h at 800 °C.

**Figure 3:**
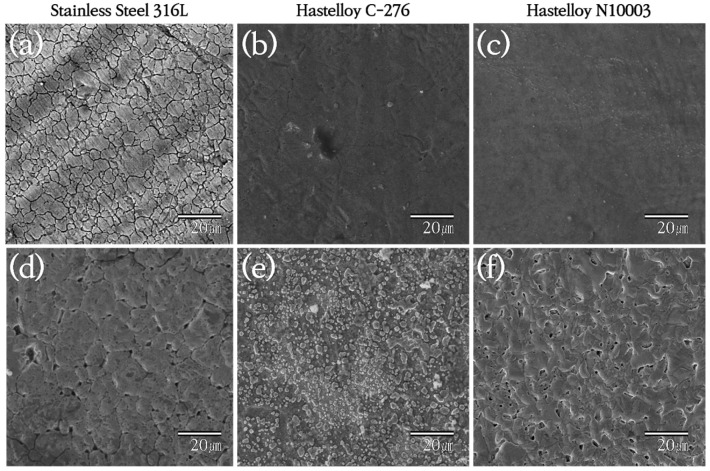
SEM images of structural materials (**a**–**c**) before and (**d**–**f**) after immersion in ClNaK salt for 48 h at 800 °C.

**Figure 4:**
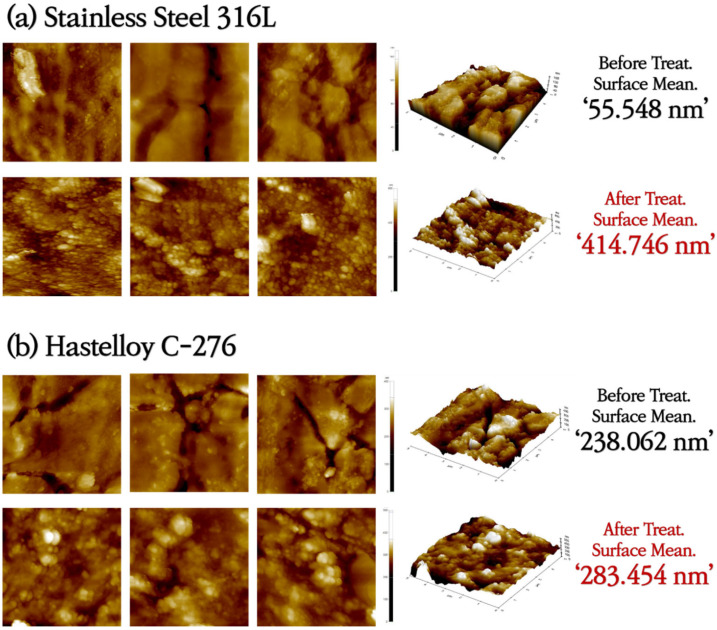

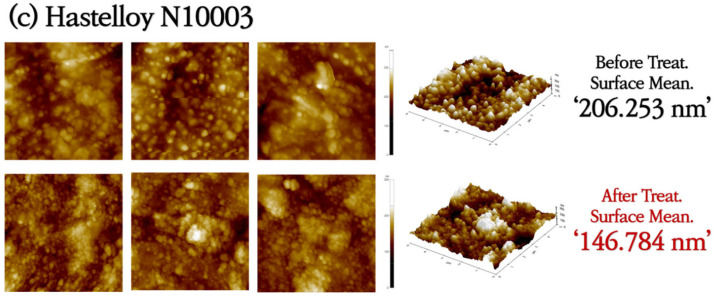
AFM measurement results for surface mean thickness and grain boundary changes before and after ClNaK salt immersion corrosion reaction and 3D surface roughness mapping indices. The images in the top (bottom) row for each alloy correspond to the AFM results obtained before (after) the corrosion test.

SS316L has a native Cr_2_O_3_ oxide protective layer, while C-276 and N10003 have dense Ni–Mo–Cr layers instead of metal oxides on their surfaces. Ni–Mo–Cr is a corrosion-preventing layer similar to Cr_2_O_3_. However, the reaction of the metal ions on the surfaces with molten salt causes the protective layers to disappear. Elements with low Gibbs free energy react preferentially with Cl and are eluted. As shown in Figure 1, the reactions occur in the order of Cr > Fe > Ni > Mo [23]. The alloy changes after the corrosion of the structural materials in the ClNaK eutectic salts were visually observed (Figure 2). Figure 3 shows SEM images of the surface grain shapes in the three alloy candidates at 1000× magnification. Figure 3d–f shows that the surface grain size changed considerably after the corrosion test. In addition, C-276 in Figure 3e exhibited an irregular surface state without distinct surface grain boundaries. Because corrosion begins with damage to the surface, this result can be used to predict the degree of corrosion in advance. Owing to the challenges in obtaining information about the entire sample from SEM results, the mean surface thicknesses before and after the corrosion of nine substrates were determined using AFM. The changes in the surface roughness are listed with five significant figures in Figure 4, and the mean value of the nine substrates is listed with six significant digits. Figure 4a shows that the surface thickness of SS316L increased by approximately 8 times from 55 to 414 nm. Compared to the SEM results, more grain boundaries were present on the surface. This proves that different reactions occurred as the substrate surface was being damaged and that these reactions covered the surface. In contrast, Figure 4b shows that the mean surface thickness of C-276 increased slightly by 15% and grain aggregation occurred, while Figure 4c shows that the surface thickness of N10003 decreased from 206 to 146 nm, proving that the surface grain was damaged and eluted as molten salt.

### 3.2. Corrosion Rate of Structural Materials in ClNaK Salt

**Figure 5:**
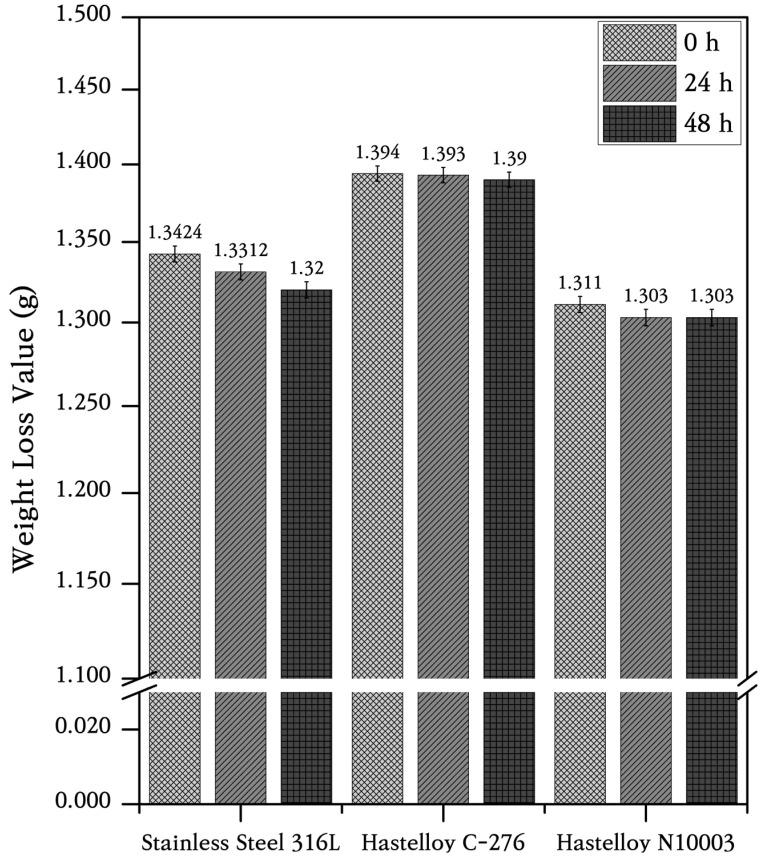
Weight loss of structural material candidates after ClNaK salt immersion test. (Weight reduction in SS316L after 24 h: 0.834% and 48 h: 1.668%. Weight reduction in C-276 after 24 h: 0.071% and 48 h: 0.286%. Weight reduction in N10003 after 24 h: 0.61% and 48 h: 1.22%.).

It was reported that the lifecycles of structural materials can be predicted from their mass loss and corrosion rates measured over long periods of several years (mmpy, millimeter penetration per year) [24,25]. Figure 5 shows the mass changes calculated based on the averages of nine alloy samples with 0.005 g error bars. Only SS316L lost mass after corrosion at high temperatures in an atmospheric environment after both 24 and 48 h. The corrosion rates of Ni and Fe alloy metals are given by

(1) $$mmpy\;\left(\frac{mm}{year}\right)=KW/ATD.\;K:factor\;(8.76\times 10^{4}\;mm/y)\;W:sample\;mass\;loss\;during\;corrosion\;(g)\;A:sample\;area\;({cm}^{2})\;T:sample\;exposure\;time\;(h)\;D:sample\;density\;(g/{cm}^{3})$$

Using Equation (1), the yearly corrosion rates of SS316L, C-276, and N10003 were obtained as 5.268, 0.8211, and 1.647 mmpy, respectively. These corrosion rates show that the main elements in the structural materials were lost through chloride formation. The corrosion rate can also be calculated using the ion activation acceleration coefficients of Cr, Fe, and Ni, which are the main elements present in the alloys. A formula based on the activation energy and acceleration factor was used to evaluate the stability of the alloys based on their reactivity with chlorine during corrosion. The chlorine compounds in the Ni and Fe alloy metals were formed through the reactions listed below. The acceleration factor can be obtained from the maximum activation energy amongst Fe, Cr, Mo, Ni, and other elements using Equation (2). Cr^3+^, Ni^2+^, and Fe^2+^ ions react with Cl in an atmospheric environment containing O_2_ and H_2_O. The compounds formed upon ion desorption react readily with O in an unshielded air environment. Ion elution occurs through chlorination, as listed in the following reactions:

(2) $$M+2{Cl}^{-}\to M{Cl}_{2}+2e$$

(3) $$M+3{Cl}^{-}\to M{Cl}_{3}+3e$$

(4) $$M{Cl}_{2}\to M^{2+}+2{Cl}^{-}$$

(5) $$M{Cl}_{3}\to M^{3+}+3{Cl}^{-}$$

The Swedish chemist Svante August Arrhenius proposed an equation that relates the reaction rate to the activation energy and temperature generated when a liquid, gas, or solid reacts chemically [26]. The reaction rates and Arrhenius rate constants were utilized in the correlation formula between ion movement and the temperature to determine the internal energy and ion migration activity of ClNaK at the eutectic point of 657 °C and _Δ_T of 150 °C with 800 °C ClNaK. The higher ion migration rates at 800 °C, which is above the melting point of 657 °C, led to higher corrosion, as indicated by the formula that relates the corrosion strength to the rate constant [27]. The Arrhenius equation gives the velocity of matter as a function of temperature:

(6) $$k\left(T\right)=A\exp\left(-\frac{E_{a}}{RT}\right),$$

where *k*(*T*) is the reaction rate (1/s), *A* the Arrhenius velocity constant (pre-exponential factor), *E_a_* the activation energy (J/mol), *T* the absolute temperature (K), and *R* the gas constant (8.314 J/mol·K). The Arrhenius model is the most widely used acceleration model for temperature tests [28,29]. Equation (6) implies that as the gas temperature increases, the number of molecules in an unstable state increases along with the molecular activity and the number of collisions between molecules. Therefore, increasing the temperature of the metal in a reactor increases the reaction rate and induces latent deformation in the reactor. A temperature-specific heat model can be constructed by combining the Arrhenius and inverse models. Using the principle that the lifetime in the Arrhenius model is proportional to the reciprocal of the reaction rate [30], the following equation can be derived:

(7) $$AF=C\cdot e^{\frac{B}{T}}=C\cdot e^{\frac{E_{a}}{k\cdot T}}={exp}^{\left\{\frac{E_{a}}{k}\cdot \left(\frac{1}{T_{op}}-\frac{1}{T_{expt}}\right)\right\}},\;$$

where *AF* is the acceleration factor (structural material acceleration life), *C* is a structural material model constant, *B* = *E_a_*/*k* (*T*), *k* is Boltzmann’s constant (8.623 × 10^−5^ eV/K), *T_expt_* is the experimental temperature, and *T_op_* is the operating temperature. When temperature and specific thermal stress (temperature, load, pressure, or density) are applied simultaneously as accelerating stress factors, the temperature increases exponentially instead of linearly [31]. This implies that the temperature effects in corrosion immersion experiments performed at 800 °C are accelerated by 5.792 times compared to those at the MSR operating temperature of 650 °C. In other words, performing a corrosion test at 800 °C for 48 h can achieve similar effects to performing the corrosion test at 650 °C for 278 h. This is because NaCl–KCl mixed salt at 650 °C does not contain trivalent or tetravalent chloride uranium or plutonium and therefore cannot be used in a liquid state [23]. Equation (7) is a theoretical expression. It is expected that the experimental results for Fe or Ni-based alloys will be more significant for elucidating the corrosion dynamics than the theoretical calculation results for corrosion for 278 h at 650 °C. Because the calculated acceleration constant increases exponentially with the temperature difference, the internal cross-sections of the structural alloys were observed to confirm their corrosion depths and shapes to verify the corrosion mechanism.

### 3.3. Cross-Section Invasion of Cl-Based Salt

**Figure 6:**
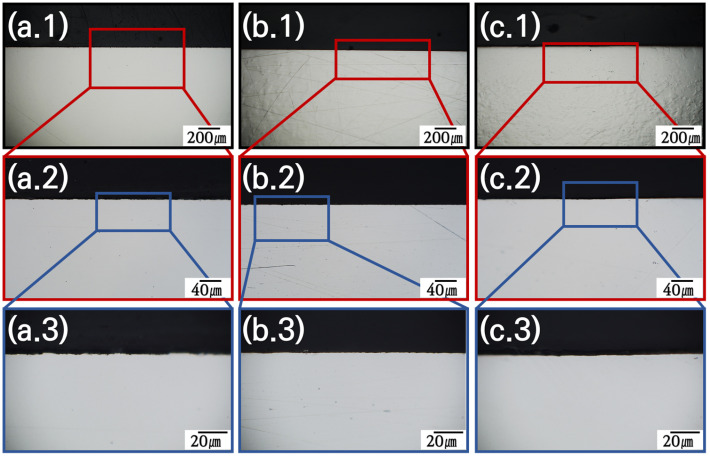
OM images of polished alloy cross-sections before salt immersion test. Structural material candidates: (**a**) SS316L, (**b**) C-276, and (**c**) N10003. Magnification (**1**): ×140, (**2**): ×700, and (**3**): ×2000.

**Figure 7:**
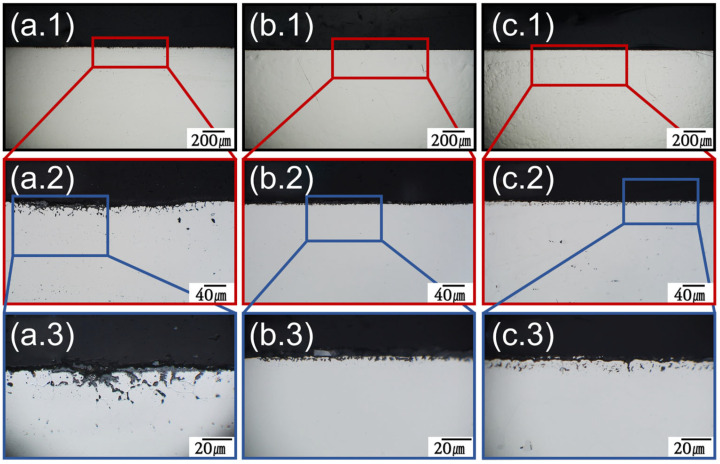
OM images of polished alloy cross-sections after salt immersion test. Structural material candidate: (**a**) SS316L, (**b**) C-276, and (**c**) N10003. Magnification: (**1**): ×140, (**2**): ×700, and (**3**): ×2000.

**Figure 8:**
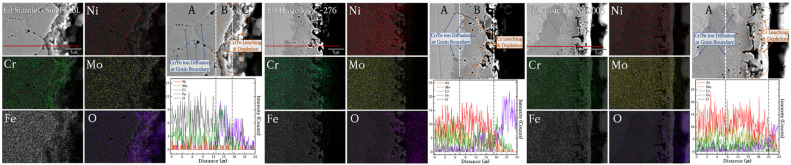
Cross-sectional SEM–EDX results for (**a**) SS316L, (**b**) C-276, and (**c**) N10003. SEM–EDX was performed to analyze the corrosion behavior of the Cl/oxide film surface protective layers in the structural materials and determine the element distributions at the interfaces after corrosion. Zone A: matrix (bulk), Zone B: corrosion diffusion layer, and Zone C: outside (epoxy). Red line: 24 μm line scan section.

Grain boundaries provide the optimal pathways for the movement of bulk ions to the surface. The ×2000 magnification OM image in Figure 6 shows that the interior of the alloy had a smooth cross-section before the molten salt reaction. After corrosion, SS316L was corroded from the surface to the bulk as shown in Figure 7(a.1–a.3). Figure 7(a.3) shows that the 6–10 μm thick surface protective layer was damaged, and an oxidation-corrosion layer was formed, and that corrosion began to penetrate into the bulk in the shape of a tree branch. In comparison, in the corresponding results for C-276 and N10003 shown in Figure 7(b.3) and Figure 7(c.3), respectively, surface corrosion was induced, but deep penetration did not occur, and grain boundaries can be observed in the OM images. SEM–EDX analysis of the cross-sections was performed to confirm the exact thickness of the corrosion layer and determine the distribution of Ni, Mo, and Cr at the interface between the surface and matrix based on the thickness of the oxide corrosion film (Figure 8). Significant element loss was observed at the interfaces between the surfaces and the matrices of the candidate alloys. Figure 8a shows the degree of corrosion in the Fe-based alloy. The triple point of the grain boundary was located in the corrosion sublayer and identified as a diffusion path for Fe and Cr. The corrosion diffusion distance corresponding to Zone B in Figure 8a was approximately 30 μm. The SEM–EDX results indicate sporadic regions with interfacial pitting corrosion in the alloys. The line EDX results indicate the quantities of the imaged elements. However, because of the heavy noise present, only the general trends are referred to in the following. In SS316L, in which sporadic pitting corrosion was evident, the density of the main elements decreased with decreasing distance from the surface because of the elution of Cr and Fe ions. Figure 8b shows the SEM–EDX results for corroded C-276. The grain length and size in the section with the lowest density were determined. Line-EDX was performed on the protective diffusion barrier layer, and Zone B was determined to be approximately 10 μm thick. This made it possible to observe significant changes in the pore layer and the grain boundaries of the rapidly collapsed ions on the surface. However, Ni elution did not induce changes in the Ni and Mo ions in Zone B as rapid as those in Zone A. Zone A corresponds to the characteristic Ni–Mo–Cr layer of C-276, which prevented internal penetration. SS316L and C-276 were selected to confirm the effects of the Cr content on the corrosion behavior. Figure 8c confirms that there was a large diffusion distance from the Cl and O corrosion layer to the protective layer. Large voids that penetrated deeply into the material are visible. The grain boundary diffusion distance was found to reach up to 20 μm. It is challenging to distinguish Zones A, B, and C in N10003, and there was lower internal pitting corrosion compared to that in C-276.

### 3.4. Selection of Structural Materials for Cl-Based MSRs

**Figure 9:**
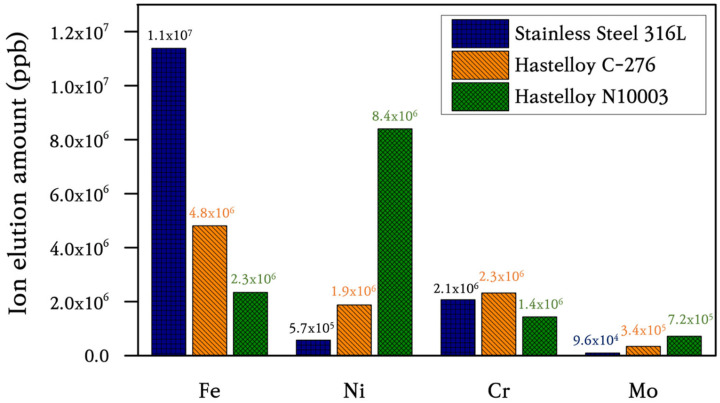
Results of ICP–MS analysis to determine amount of elemental and ion elution in candidate alloys after corrosion reaction.

**Figure 10:**
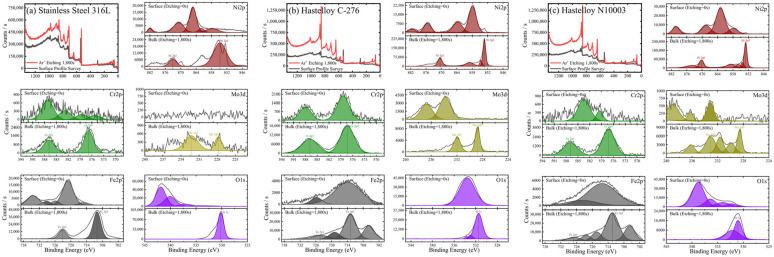
XPS analysis results for Ni, Cr, Mo, Fe, and O contents in (**a**) SS316L, (**b**) C-276, and (**c**) N10003. Deep etching sputtering was used to determine the differences between the elements present on the surface and in the bulk from the depth profile surface. Plasma etching was performed from 0 to 1800 s.

**Figure 11:**
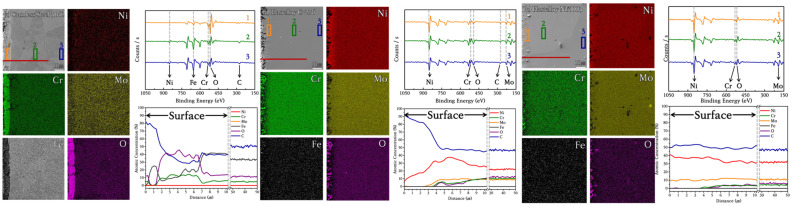
Cross-section profiles (spots 1, 2, and 3) obtained using AES, elemental mapping, and small area analysis. Line scanning (red line: 50 μm) using Auger electrons with excellent depth resolution and surface sensitivity: (**a**) SS316L, (**b**) C-276, and (**c**) N10003.

The suitability of the candidate substrates was evaluated by measuring the extent of elution in the main protective layer comprising Ni, Mo, Cr, and Fe ions using ICP–MS. Significant amounts of ions were eluted from SS316L and N10003. By-products were formed when Fe, Ni, and Cr ions in the structural materials were exposed to Cl ions in an H_2_O environment and high temperatures. The reactions between the metallic and Cl ions occurred together with the internal migration and diffusion of ions through grain boundaries. The subsequent removal of the Cr oxide film or Ni–Mo–Cr protective layer resulted in the exposure of iron or nickel ions to the molten salt and their depletion. The results in Figure 9 show that bulk Fe and Ni were partially lost and eluted as salts. Mo of 95.83, 344.62, and 720.44 ppm were eluted in SS316L, C-276, and N10003, respectively. The largest amounts of Ni, Mo, and Cr were eluted from N10003. The amount of nickel eluted was determined by the differences between Ni-based alloys. The Ni–Mo–Cr surface protective layer prevented the elution of ions to the outside. The large amount of Ni eluted in N10003, in which Ni constitutes the major component, provides evidence for the removal of the surface protective layer. In contrast, the eluted amounts of Ni, Mo, and Cr shown in Figure 9 indicate that a dense protective layer was retained in C-276. Figure 10a–c shows the quantitative analysis XPS results for the elemental compositions from the surface to the inner bulk after the oxide film/protective layers were removed using Ar sputtering [32]. The differences between the internal and initial elemental compositions on the surface provide information on the types of reactions that occurred during corrosion [33]. The kinetic energy provides information on the amounts of elements at different locations in the sections and on the surface. The internal characteristics were determined over a sputtering time of 1800 s. The elemental composition from 0 to 100 s followed the sequence of Cr > Ni > Fe, and the concentration of O decreased as the sputtering time increased. To determine the cross-sectional element distribution more accurately, EDX was performed, and the variation in the distribution with the depth was measured using the M, N, L, and K angles of the outermost O, Cr, Mo, Ni, and Fe electron layers. The profiles before and after corrosion at the same depths were measured to determine the element losses at different thicknesses. Figure 10a shows the XPS spectrum obtained after the corrosion of the Fe-based alloy. O was not detected on the surface, and 87.39% Fe (530.94 eV) > 12.02% Ni (710.41 eV) > 0.30% Mo (855.36 eV), 0.30% Cr (577.11 eV) were detected. In particular, in the bulk, 65.69% O > 20.01% Fe > 13.7% Ni > 2.81% Cr > 0.46% Mo were detected. Corrosion had no effect on the 16% Cr content on the surface and bulk in SS316L. A similar trend was also observed in the separated peaks of the Ni-based alloys. The elemental compositions detected on the surface of C-276 are 87.74% O > 11.05% Ni > 0.58% Mo > 0.21% Cr [Figure 10b]. These results show that corrosion did not penetrate into the surface and anti-corrosion layers, and the alloy interior. The left shifts in the peaks for each element after the oxidation of C-276 resulted in slight differences between the elemental compositions of C-276 and N10003. However, the Fe-based alloys exhibited significant element losses in chlorine salt environments. It is thus concluded that SS316L was more oxidized than the Hastelloy structural alloy. In the XPS results shown here, most of the alloy atoms were detected in the bulk layer [Figure 10a–c]. This shows that most of the elements were not present on the surface. After sputtering for 1800 s, the peak exhibited a slight shift relative to that before corrosion, and many elements were detected as the removal depth increased from the surface layer (corrosion layer) to the barrier layer (ion diffusion layer) under long etching times. The changes in the element ratios before and after etching are listed in Table 2. Large amounts of Fe, Cr, and Ni were observed in the surface layer, while a depleted element ratio was observed in the diffusion layer. This proves that the elements that diffused through the grain boundary were more abundant in the surface and internal bulk compared to those in the diffusion layer. To cross-validate this conclusion, XPS point profiling was performed on the interface using AES. Figure 11 shows the results for elemental line analysis performed simultaneously with elemental mapping. Regions as small as approximately 20 nm could be resolved using Auger electrons. XPS point profiling was performed on specified points on the (1) outer, (2) intermediate, and (3) bulk layers to obtain the data shown in Figure 10. In addition, because AES is a more sensitive technique than SEM–EDX, which was used to obtain the results shown in Figure 8, the changes in internal grain boundaries and element counts can be observed. The AES analysis, therefore, complements both the XPS and SEM–EDX analyses. The element distribution ratios in the surface and bulk shown in Figure 11a are very similar to those in the XPS spectrum in Figure 10. However, the Fe peak intensity in SS316L is lower, and a large amount of O was detected on the surface, and the bulk Fe-O compound content [(XPS spots 2 and 3 in Figure 11a)] was maintained. The Ni and Mo peak intensities in C-276 and N10003 were not changed significantly. Figure 11b,c shows that C-276 and N10003, which are considered to be suitable with respect to molten salt under air and humidity conditions, were hardly corroded under molten salt conditions without any purity treatment. The increased Ni, Mo, and Cr contents in the interior inhibited the continuous penetration of molten salt. In particular, N10003 exhibited the most outstanding characteristics among the candidate alloys with respect to the number of atoms and the similarity of the element ratios between the surface and the diffusion layer. Figure 11a shows that in SS316L, severe elution occurred up to approximately 9 μm, and a stable inner layer was evident after 30 μm. The depletion mechanism of the surface corrosion protection layer comprises the removal of the protective layer on the surface by molten salt with impurities and the subsequent penetration of the molten salt into the interior or the initiation of ion elution from the inside. Surface grain boundary changes were observed in SS316L and N10003 (Figure 3), although no significant elemental elution occurred at the N10003 interface (Figure 11).

## 4. Discussion

### 4.1. Fe-Balance vs. Ni-Balance Alloys

SS316L, which has been selected as the structural material in many applications [34,35], was introduced in this study. As a typical Fe-based austenitic alloy, corrosion is prevented in SS316L through the formation of chromium oxide on the surface [36]. Chromium oxide is used in various antioxidants but is reportedly vulnerable in alkaline and corrosive environments at high temperatures [37]. Meanwhile, Fe is known to have better reactivity with halogen ions than Ni [35]. The results in Figure 7, Figure 8 and Figure 11 show that the Fe-based alloys have the same corrosion tendencies and are unsuitable for corrosive unpurified chlorine-based salt environments. At the same time, Hastelloy, which is a representative Ni superalloy structural material, has attracted much attention for applications in molten salt nuclear power plants [38,39]. Owing to their high-temperature creep properties and corrosion resistance, C-276 and N10003 have been identified as suitable substrate candidates for MSRs [40]. It was reported that continuous ion exposure due to surface vacancies is relatively insignificant in these alloys owing to the formation of stable and dense surface layers containing Ni–Mo–Cr [41,42]. Figure 3 and Figure 11 also show the same trends for ion depletion due to surface damage caused by exposure to high-temperature molten salt. The alloys used in this study are commonly utilized in existing nuclear power plants. This indicates that the alloy used is not significantly impacted by radiation exposure. Based on these observations, a comparison of the Fe- and Ni-based alloys indicates that the Ni-based alloy exhibits greater stability against molten salt corrosion. Therefore, it was concluded that SS316L is unsuitable for use as a structural material.

### 4.2. Cr Structural Materials 16.0 wt% Cr vs. 7.0 wt%

Chromium is well known for its antioxidant properties. The oxidation resistance of alloys can be improved by increasing their Cr content. High-Cr steel alloys are used in structural materials that are particularly vulnerable to corrosion. However, it was also reported that structural materials with high Cr contents are unsuitable for MSRs [43,44]. Because Cr precipitates quickly in molten salt, alloys with lower Cr contents are more stable in molten salt, especially in chlorine-based environments [45]. Therefore, alloys with low Cr content are recommended for applications involving chlorine-based molten salts [46]. However, in this study, we found that C-276, which has a higher Cr content, is more stable against corrosion immersion than N10003. We attribute the corrosion resistance of Cr to its stability against moisture and oxygen in unpurified salt [47,48]. Although SS316L has a chromium oxide film, which may prevent corrosion [49], the chromium oxide does not significantly hinder corrosion in a high-iron content and high-temperature chlorine-based environment [50]. In other words, the chromium oxide layer does not serve as an adequate anti-corrosion layer under immersion in unrefined 0.506 NaCl–0.494 KCl molten salt at 800 °C for 48 h. In comparison, Hastelloy is suitable for environments requiring high-temperature creep properties because its stable Ni–Mo–Cr surface inhibits compound reactions at high temperatures [51]. C-276 and N10003, which have the respective compositions of 16Cr-Ni and 7Cr-Ni, share the same metal oxides and corrosion prevention mechanisms and differ only in their Cr contents. This content difference leads to different levels of stability in the surface grain boundaries after corrosion in environments containing moisture and oxygen, as shown in Figure 3. In these results, C-276 showed more stable corrosion compared to N10003 due to its high Cr content and Ni-based alloy. It can thus be concluded that a higher Cr content led to the formation of a denser surface Ni–Mo–Cr layer [52]. The calculated corrosion rate for high-Cr Ni alloys in unpurified molten salt corrosive environments is only half that of low-Cr Ni alloys.

## 5. Conclusions

In this study, the corrosion behavior of structural materials in an unrefined chlorine-based molten salt environment was investigated. SS316L, C-276, and N10003 were selected as candidates for structural materials, and the reason for the selection is to compare Fe-based alloy and Ni-based alloy, and to compare high Cr ratio alloys and low Cr ratio alloys. The corrosion rates of SS316L, C-276, and N10003 were found to be 5.268, 0.821, and 1.647, respectively. The observed corrosion patterns in the surface, diffusion, and bulk layers of the structural materials indicate that their corrosion mechanism comprises the four stages of (1) destruction of the protective layer after surface pitting by molten salt, (2) movement of diffusion layer ions to the surface layer, (3) elution of large quantities of ions to grain boundaries due to exhaustion of mobile ions, and (4) penetration of corrosive substances including impurities into the interior structure of the alloy and its resultant degradation. Through the evaluation of the properties after the corrosion experiment of the alloy, it was confirmed that the corrosion resistance characteristics of Hastelloy C-276 were the best. These results show that in a chlorine-based molten salt environment, Ni-based alloys have better corrosion resistance than Fe-based alloys and high Cr contents than low Cr contents alloys. To cross-validate, it is essential to observe the high-temperature corrosion behavior in a purified chlorine-based molten salt environment. Further studies are underway to confirm the high-temperature corrosion behavior of chlorine-based molten salts using purified salts in an inert environment where moisture and oxygen are controlled and will be reported in subsequent studies.

## Figures and Tables

**Table 1:** Types and amounts of structural alloy elements.

Structural Material/ Element Ratio (wt%)	Stainless Steel 316L (Fe-Based Alloy)	Hastelloy C-276 (Ni-Based Alloy)	Hastelloy N10003 (Ni-Based Alloy)
Fe	Balance	5.0	4.0
Ni	10.0	Balance	Balance
Cr	16.0	16.0	7.0
Mo	2.0	16.0	16.0
Mn	2.0	1.0	0.8
Si	0.75	0.08	1.0
C	0.03	0.01	0.06
Cu	-	0.5	0.35
Co	-	2.5	0.02
W	-	4.0	0.5
V	-	0.35	0.5
N	0.1	-	-
P	0.045	-	-
S	0.03	-	-

**Table 2:** Elemental change ratios before/after XPS depth profile (at%).

		Ni 2p1 (870 eV)	Cr 2p1 (584 eV)	Mo 3d3 (231 eV)	Fe 2p1 (720 eV)	O 1s (531 eV)
		Ni 2p3 (853 eV)	Cr 2p3 (575 eV)	Mo 3d5 (228 eV)	Fe 2p3 (707 eV)	
SS316L	Surface (at%)	11.22	-	0.03	87.39	-
		0.80	0.30	0.27	0	
	* Bulk (at%)	4.01	0.18	-	7.15	65.69
		9.69	0.43	-	12.86	
Hastelloy C	Surface (at%)	3.89	-	0.58	0.20	87.74
		7.16	0.21	-	0.23	
	Bulk (at%)	38.86	1.15	0.28	-	18.86
		36.31	1.66	0.18	2.70	
Hastelloy N	Surface (at%)	-	0.06	-	7.44	84.38
		7.59	0.46	-	0.08	
	Bulk (at%)	38.46	0.18	0.69	-	17.22
		39.22	0.27	0.19	3.76	

* Bulk refers to the depth profile after Ar plasma etching for 1800 s.

## Data Availability

The raw data supporting the conclusions of this article will be made available by the authors upon request.

## References

[1] Lenzen M. (2008). Life cycle energy and greenhouse gas emissions of nuclear energy: A review. Energy Convers. Manag..

[2] Buongiorno J., Corradini M., Parsons J., Petti D. (2019). Nuclear energy in a carbon-constrained world: Big challenges and big opportunities. IEEE Power Energy Mag..

[3] Jo H.J., Yeom H., Gutierrez E., Sridharan K., Corradini M. (2019). Evaluation of critical heat flux of ATF candidate coating materials in pool boiling. Nucl. Eng. Des..

[4] Kim H.G., Kim I.H., Jung Y.I., Park D.J., Park J.H., Yang J.H., Koo Y.H. Progress of surface modified Zr cladding development for ATF at KAERI. Proceedings of the 2017 Water Reactor Fuel Performance Meeting.

[5] Kim H.G., Kim I.H., Park J.Y., Koo Y.H. (2015). Application of coating technology on zirconium-based alloy to decrease high-temperature oxidation. Zirconium in the Nuclear Industry: 17th International Symposium.

[6] Kim H.G., Yang J.H., Kim W.J., Koo Y.H. (2016). Development Status of Accident-Tolerant Fuel for Light Water Reactors in Korea. Nucl. Eng. Technol..

[7] Kim D.Y., Lee Y.N., Kim J.H., Kim Y., Yoon Y.S. (2020). Applicability of swaging as an alternative for the fabrication of accident-tolerant fuel cladding. Energies.

[8] Kim J.-W., Min H.-W., Ko J., Kim Y., Yoon Y.S. (2022). Study of structural stability at high temperature of pseudo-single tube with double layer as an alternative method for accident-tolerant fuel cladding. J. Nucl. Mater..

[9] Xia S.Q., Wang Z., Yang T.F., Zhang Y. (2015). Irradiation Behavior in High Entropy Alloys. J. Iron Steel Res. Int..

[10] Kim M.S., Lee S.H., Jung J.G., Eah K. (2021). Prediction of grain structure in direct-chill cast Al–Zn–Mg–Cu billets using cellular automaton-finite element method. Prog. Nat. Sci. Mater. Int..

[11] Tan L., Allen T.R., Busby J.T. (2013). Grain boundary engineering for structure materials of nuclear reactors. J. Nucl. Mater..

[12] Lee H.G., Kim D., Kim W.J., Park J.Y. (2022). Reaction–diffusion bonding of CVD SiC using CrAl thin coating layer. J. Korean Ceram. Soc..

[13] Romatoski R.R., Hu L.W. (2017). Fluoride salt coolant properties for nuclear reactor applications: A review. Ann. Nucl. Energy.

[14] Rosenthal M. (2010). An Account of Oak Ridge National Laboratory’s Thirteen Nuclear Reactors. http://info.ornl.gov/sites/publications/files/Pub20808.pdf.

[15] McFarlane J., Taylor P., Holcomb D., Poore W.P. (2019). Review of Hazards Associated with Molten Salt Reactor Fuel Processing Operations. www.osti.gov.

[16] Galashev A.Y. (2022). Molecular dynamics study of ionic diffusion and the FLiNaK salt melt structure. Nucl. Eng. Technol..

[17] Karfidov E., Nikitina E., Erzhenkov M., Seliverstov K., Chernenky P., Mullabaev A., Tsvetov V., Mushnikov P., Karimov K., Molchanova N. (2022). Corrosion Behavior of Candidate Functional Materials for Molten Salts Reactors in LiF–NaF–KF Containing Actinide Fluoride Imitators. Materials.

[18] Creasman S.E., Pathirana V., Chvala O. (2023). Sensitivity study of parameters important to Molten Salt Reactor Safety. Nucl. Eng. Technology.

[19] Guo S., Zhang J., Wu W., Zhou W. (2018). Corrosion in the molten fluoride and chloride salts and materials development for nuclear applications. Prog. Mater. Sci..

[20] Qu L., Wang Q., Mao J., Xu S., Zhang H., Shi Z., Li X. (2021). Study of anti-chlorine corrosion of anion exchange resin based superhydrophobic cement mortar in chloride salt environment. Constr. Build. Mater..

[21] He Z., Zhao H., Song J., Guo X., Liu Z., Zhong Y., Marrow T.J. (2022). Densification of matrix graphite for spherical fuel elements used in molten salt reactor via addition of green pitch coke. Nucl. Eng. Technol..

[22] Sadiq M., Wen F., Dagestani A.A. (2022). Environmental footprint impacts of nuclear energy consumption: The role of environmental technology and globalization in ten largest ecological footprint countries. Nucl. Eng. Technol..

[23] Capelli E., Beneš O., Konings R.J.M. (2015). Thermodynamic assessment of the LiF-ThF4-PuF3-UF4 system. J. Nucl. Mater..

[24] Wang Y., Goh B., Nelaturu P., Duong T., Hassan N., David R., Moorehead M., Chaudhuri S., Creuziger A., Hattrick-Simpers J. (2021). Accelerated Discovery of Molten Salt Corrosion-Resistant Alloy by High-Throughput Experimental and Modeling Methods Coupled to Data Analytics. arXiv.

[25] Persdotter A., Eklund J., Liske J., Jonsson T. (2020). Beyond breakaway corrosion—Influence of chromium, nickel and aluminum on corrosion of iron-based alloys at 600 °C. Corros. Sci..

[26] Logan S.R. (1982). The Origin and Status of the Arrhenius Equation. https://pubs.acs.org/sharingguidelines.

[27] Kang M.J., Yoon D.H. (2022). Effects of impurities on the slip viscosity and sintered properties of low-soda easy-sintered α-alumina. J. Korean Ceram. Soc..

[28] Yang G. (2007). Life Cycle Reliability Engineering.

[29] Sopher R., Nixon J., Gorecki C., Gefen A. (2011). Effects of intramuscular fat infiltration, scarring, and spasticity on the risk for sitting-acquired deep tissue injury in spinal cord injury patients. J. Biomech. Eng..

[30] Peleg M., Normand M.D., Corradini M.G. (2012). The Arrhenius equation revisited. Crit. Rev. Food Sci. Nutr..

[31] Li X., Zhang Y., Yue B., Yan L., Jiang T., Peng S. (2020). Unifying the diffusion coefficients of lanthanides and actinides in binary molten salt mixtures: A data review. J. Mol. Liq..

[32] Phan T.T.T., Nguyen T.D., Lee J.S. (2022). Vacuum plasma treatment on carbon nanoparticles for highly sensitive square wave voltammetric sensor of heavy metal ions. Synth. Met..

[33] Lee S.R., Bae K.M., Baek J.J., Kang M.C., Lee T.I. (2021). Adhesion enhancement between aluminum and butyl rubber by (3-mercaptopropyl) trimethoxy silane for vibration damping plate. J. Adhes. Sci. Technol..

[34] Kondo M., Nagasaka T., Xu Q., Muroga T., Sagara A., Noda N., Ninomiya D., Nagura M., Suzuki A., Terai T. (2009). Corrosion characteristics of reduced activation ferritic steel, JLF-1 (8.92Cr-2W) in molten salts Flibe and Flinak. Fusion Eng. Des..

[35] Raiman S.S., Lee S. (2018). Aggregation and data analysis of corrosion studies in molten chloride and fluoride salts. J. Nucl. Mater..

[36] Lei Y.B., Wang Z.B., Zhang B., Luo Z.P., Lu J., Lu K. (2021). Enhanced mechanical properties and corrosion resistance of 316L stainless steel by pre-forming a gradient nanostructured surface layer and annealing. Acta Mater..

[37] Li X., Yang J., Feng X., Hu Y., Zou H., Zhang C., Xiong L., Zheng X., Liu Y. (2020). Electrochemical performance of porous Ni-Cr-Mo-Cu alloys for hydrogen evolution reactions in alkali solution. Mater. Res. Express.

[38] Luo Y., Jiang W., Zhang Y., Hao M., Tu S.T. (2018). Creep rupture behavior of Hastelloy C276-BNi2 brazed joint. Mater. Sci. Eng. A.

[39] Patel N.S., Pavlík V., Boča M. (2017). High-Temperature Corrosion Behavior of Superalloys in Molten Salts—A Review. Crit. Rev. Solid State Mater. Sci..

[40] Zhu H., Muránsky O., Wei T., Davis J., Budzakoska-Testone E., Huang H., Drew M. (2021). The effect of applied stress on the high-temperature creep behaviour and microstructure of NiMoCr Hastelloy-N^®^ alloy. Materialia.

[41] Danon A.E., Muránsky O., Karatchevtseva I., Zhang Z., Li Z.J., Scales N., Kruzic J.J., Edwards L. (2020). Molten salt corrosion (FLiNaK) of a Ni–Mo–Cr alloy and its welds for application in energy-generation and energy-storage systems. Corros. Sci..

[42] Muránsky O., Yang C., Zhu H., Karatchevtseva I., Sláma P., Nový Z., Edwards L. (2019). Molten salt corrosion of Ni-Mo-Cr candidate structural materials for Molten Salt Reactor (MSR) systems. Corros. Sci..

[43] Olson L.C., Ambrosek J.W., Sridharan K., Anderson M.H., Allen T.R. (2009). Materials corrosion in molten LiF-NaF-KF salt. J. Fluor. Chem..

[44] Qiu J., Zou Y., Yu G., Liu H., Jia Y., Li Z., Huai P., Zhou X., Xu H. (2014). Compatibility of container materials with Cr in molten FLiNaK salt. J. Fluor. Chem..

[45] D’Souza B., Zhuo W., Yang Q., Leong A., Zhang J. (2021). Impurity driven corrosion behavior of HAYNES^®^ 230^®^ alloy in molten chloride Salt. Corros. Sci..

[46] Ding W., Gomez-Vidal J., Bonk A., Bauer T. (2019). Molten chloride salts for next generation CSP plants: Electrolytical salt purification for reducing corrosive impurity level. Sol. Energy Mater. Sol. Cells.

[47] Ouyang F.Y., Chang C.H., You B.C., Yeh T.K., Kai J.J. (2013). Effect of moisture on corrosion of Ni-based alloys in molten alkali fluoride FLiNaK salt environments. J. Nucl. Mater..

[48] Yang X., Liu M., Gao Y., Zhang D., Feng S., Liu H., Yu G., Wu G., Wang M., Zhou X. (2016). Effect of oxygen on the corrosion of SiC in LiF-NaF-KF molten salt. Corros. Sci..

[49] Bae J.H., Yu J.M., Dao V.H., Lok V., Yoon K.B. (2021). Effects of processing parameters on creep behavior of 316L stainless steel produced using selective laser melting. J. Mech. Sci. Technol..

[50] Yu J.M., Dao V.H., Yoon K.B. (2020). Investigation of creep behavior of 316L stainless steel produced by selective laser melting with various processing parameters. J. Mech. Sci. Technol..

[51] Cheng W.J., Chen D.J., Wang C.J. (2015). High-temperature corrosion of Cr-Mo steel in molten LiNO_3_-NaNO_3_-KNO_3_ eutectic salt for thermal energy storage. Sol. Energy Mater. Sol. Cells.

[52] Vignarooban K., Pugazhendhi P., Tucker C., Gervasio D., Kannan A.M. (2014). Corrosion resistance of Hastelloys in molten metal-chloride heat-transfer fluids for concentrating solar power applications. Sol. Energy.

